# Leveraging Bayesian Optimization Software for Atomic Layer Deposition: Single-Objective Optimization of TiO_2_ Layers

**DOI:** 10.3390/ma17205019

**Published:** 2024-10-14

**Authors:** Philipp Häussermann, Nikhil Biju Joseph, Daniel Hiller

**Affiliations:** 1 Institute of Applied Physics (IAP), Technische Universität Bergakademie Freiberg, 09599 Freiberg, Germany; philipp.haeussermann@physik.tu-freiberg.de; 2 Institute of Experimental Physics (IEP), Technische Universität Bergakademie Freiberg, 09599 Freiberg, Germany; nikhil-biju.joseph@student.tu-freiberg.de

**Keywords:** atomic layer deposition (ALD), silicon surface passivation, Bayesian optimization (BO), titanium dioxide (TiO_2_), process optimization

## Abstract

We demonstrate the application of free-to-use and easy-to-implement Bayesian optimization (BO) software to streamline atomic layer deposition (ALD) process optimization. By employing machine learning-based Bayesian optimization algorithms, we enhanced the silicon surface passivation quality of titanium dioxide layers deposited using titanium tetraisopropoxide (TTIP). Unlike classical designs of experimental methods, such as Box–Behnken or Plackett–Burman designs, which require a predefined set of experiments and can become resource intensive, BO offers several advantages. It dynamically updates the search strategy based on previous outcomes, allowing for efficient exploration of parameter spaces with fewer experimental runs. This adaptive approach is particularly advantageous in small-scale experiments or laboratories where time, resources, and materials are limited. In a single-objective optimization experiment, we identified constrained search spaces that limited further optimization, underscoring the importance of properly defined parameter bounds prior to the optimization process. Our findings highlight that Bayesian optimization can not only reduce time and resource costs associated with ALD process optimization but also support faster discovery of more optimal ALD process parameters, even with minimal prior knowledge of the deposition process or precursor chemistry.

## 1. Introduction

Chemical vapor deposition (CVD) processes have gained widespread acceptance as essential tools for the deposition of thin films in photovoltaic applications. Commonly utilized methods include plasma-enhanced CVD, low-pressure CVD, and atomic layer deposition (ALD)—a specialized variant of CVD where the substrate is exposed to one precursor at a time, separated by short periods of inert gas purges. The sequential nature of the ALD process and self-saturating surface reactions allow for the deposition of highly conformal coatings with precise control over layer thickness and composition, accommodating a wide range of elements and compounds for deposition [1,2,3]. These features, along with the possibility of low-temperature deposition, have made ALD of particular interest for the research and development of highly efficient solar cells, new contact materials, and novel contact structures [4,5,6,7], where precise control of thickness and composition is essential. However, the quality and properties of the deposited layers depend not only on the design of the reactor and the precursors used, but on a large number of processing parameters as well. In fact, a typical thermal ALD process involves at least seven significant parameters that affect the properties of the deposited layer: pressure, temperature, dosing times of the first and second precursor, the purge duration after each precursor step, and the number of deposition cycles. The large number of processing parameters, their unknown interaction, and the complex physics involved can make ALD process optimization a daunting task. This can be particularly problematic for small-scale experiments and laboratories, where the costs and time required to optimize the deposition of novel precursors or to achieve specific layer properties can act as substantial barriers.

Demand exists for efficient approaches to optimization that enable the fine-tuning of ALD processing parameters for arbitrary reactor designs, precursors, and optimization goals. Advances in machine learning and the increasing availability of free-to-use and easy-to-implement software packages allow for more sophisticated optimization strategies to be leveraged, potentially reducing the time and costs associated with process optimization significantly. In this work, the surface passivation quality of crystalline silicon by titanium dioxide (TiO_2_) deposited via thermal ALD was optimized by utilizing the Python software package Adaptive Experimentation Platform (Ax) (v. 0.4.3) which is published under an MIT license [8]. Ax is an accessible and general-purpose platform that allows for machine-learning-based Bayesian and bandit optimization. Using Ax, single-objective Bayesian optimization was performed for TiO_2_ layers deposited using titanium tetraisopropoxide (TTIP) and water. We note that the objective of this study is not to identify a universally optimal optimization approach for all scenarios. Instead, our aim is to highlight the potential of utilizing machine-learning-based optimization software for ALD process optimization, particularly in the context of small-scale experiments and laboratories where lengthy or complex process optimizations might be prohibitive.

## 2. Materials and Methods

### 2.1. Bayesian Optimization

Bayesian optimization (BO) is a probabilistic optimization approach that has gained considerable attention due to its ability to optimize black-box, derivative-free, and costly to evaluate functions efficiently. This versatility relies on two primary elements: a surrogate that models the objective function based on previous data, continually improving with new observations, and an acquisition function that guides the optimization process, evaluating potential points for future observation. This sequential approach offers several advantages over more common designs of experiments, like Plackett–Burman Design (PBD), used to screen for the most critical factors affecting the response, or Box–Behnken Design (BBD), used to explore the response surface [9]. Unlike BO, PBD and BBD require a predefined set of experiments, often leading to a larger number of evaluations. For instance, BBD, which is regarded as one of the most efficient and widely used designs for response surface models [10], would still require more than 50 experimental points for an optimization problem with seven factors (i.e., parameters to be optimized). Additionally, BO inherently incorporates prior knowledge about the objective function through the use of a prior distribution, typically Gaussian Processes, and actively learns from previous evaluations to propose new points that are likely to improve the objective function, reducing the number of required evaluations significantly, especially in high-dimensional spaces where classical designs would require prohibitively large sample sizes. BO models the uncertainty in unexplored regions of the parameter space, thereby guiding the search process more effectively than PBD or BBD, which do not incorporate prior beliefs or uncertainty directly. As a result, BO can explore areas of the parameter space that are more likely to contain the global optimum, even with limited data. The adaptability of BO is particularly beneficial in non-convex optimization problems with multiple local maxima (minima), where the global optimum may be found in a narrow region of the parameter space. Unlike PBD or BBD, which follow a rigid experimental design structure and do not permit dynamic adjustments to the sampling strategy, BO can shift the focus to more promising regions as new data are gathered. Moreover, statistical methods generally require completing the entire set of experiments before drawing any conclusions, while the sequential nature of BO allows for early stopping if the optimization goals are met, potentially saving both time and resources.

These advantages make BO a promising solution for optimization and design problems in a wide range of fields, including robotic locomotion [11], environmental monitoring [12], and civil engineering [13]. BO has also been used to optimize the complex physics of ALD and CVD processes, which can involve expensive precursor materials or substrates. Notably, BO has been successfully utilized in optimizing Si thin film depositions under various constraints [14], the deposition of numerous materials via ALD [15,16], as well as the post-deposition hydrogen plasma treatment of carrier-selective c-Si/SiOx/TiOx contact stacks [17].

### 2.2. Process Optimization

ALD process optimization generally focuses on reducing process cost and duration by minimizing the precursor consumption and cycle time of an already sufficiently developed deposition process. For a standard two-step (two precursor) ALD process this is achieved by minimizing the pulse times of the precursor and oxidizer as well as the purge times after each half-cycle while still achieving saturation. However, deposition processes optimized towards surface passivation quality might not necessarily operate within the ALD temperature window or saturation of the precursors used, as the processing parameters significantly affect the quality and properties of the deposited layer. This has been demonstrated for the deposition of Ga_2_O_3_ on c-Si, where layers deposited under non-saturating conditions exhibited significantly lower interface defect density ($D_{it}$) and surface saturation density $\left(J_{0s}\right)$ than saturating depositions [18]. Although no explanation was immediately apparent, it became evident that non-saturating processing parameters positively influenced the surface passivation quality of the deposited layers. The importance of deposition conditions has also been shown for Al_2_O_3_, where increased process pressure led to improved coverage and properties of films deposited using thermal ALD. This was attributed to increases in the surface saturation level and precursor diffusion as well as a higher probability of surface reactions at active sites [19]. Similarly, the surface morphology, crystallinity, uniformity, and stoichiometry of TiO_2_ layers deposited using thermal and plasma-enhanced ALD vary substantially depending on processing parameters, like temperature, pressure, and pulse times as well as the precursors used [20,21,22,23]. Accordingly, process temperatures beyond the ALD window, as well as precursor dosing and purge times outside saturation, should also be considered during the optimization of surface passivation quality. In combination with process pressure and the number of deposition cycles, seven critical processing parameters have emerged that significantly influence TiO_2_ layers deposited via thermal ALD and need to be optimized.

### 2.3. Search Space

The search space is defined by the bounds and constraints on each parameter; it encompasses the range of possible values the optimization algorithm can explore to find the optimal solution. During Bayesian optimization, candidate points can be selected from within the entire available search space. Therefore, it is advisable to impose limitations on the different parameters prior to the optimization routine, limiting the search space that needs to be considered. No or too wide boundaries unnecessarily increase the complexity of the optimization problem whereas overly narrow bounds may render the search space infeasible for complex multidimensional optimization problems. When optimizing ALD processes, the type and capabilities of the ALD reactor used impose fundamental constraints on the search space (e.g., the achievable processing temperature range depends on the system’s temperature control capabilities). In this work, the Bayesian optimization attempts were designed to assume little prior knowledge about the deposition process and the precursors used. Consequently, the search space was primarily constrained by the capabilities of the ALD system. However, to avoid excessive waste of precursor material, sensible upper limits for the dosing times of the first and second precursors were set during all experiments.

## 3. Experiment

### 3.1. Sample Preparation and Cleaning

All samples were prepared from 100 mm diameter, n-type, float-zone-grown Si-wafers with a resistivity of 1–5 Ωcm and <100> orientation. Lifetime samples were prepared by quartering double-side-polished sister wafers with a thickness of 280 ± 20 µm. Prior to deposition, all samples underwent a comprehensive cleaning process. The samples were first dipped in diluted hydrofluoric acid (HF, 2%) for 1 min before being rinsed with deionized (DI) water and subsequently immersed in a solution of DI water, hydrogen peroxide (H_2_O_2_, 35%), and hydrochloric acid (HCl, 37%) in a 7:1:1 ratio (Standard Clean 2, SC2) for 10 min at 70 °C. Afterward, the samples were etched in TMAH (25%) for 10 min at 80 °C, followed by a final SC2 cleaning step. After each intermediate cleaning step, the samples were thoroughly rinsed with DI water, dipped in diluted HF (2%) for 1 min, and rinsed again. No HF dip or DI water rinse was performed after the final SC2 step.

### 3.2. Atomic Layer Deposition

Depositions were performed via thermal ALD using a SI Atomic Layer Deposition System from Sentech Instruments GmbH (Berlin, Germany). TiO_2_ layers were deposited using titanium tetraisopropoxide (TTIP) and water with the precursor vessel heated to 80 °C. High-purity N_2_ was employed as purge and carrier gas at a constant flow rate of 120 sccm during all depositions. Samples designated for XPS measurements had TiO_2_ deposited solely on the front side, whereas samples intended for lifetime measurements received identical TiO_2_ deposition on both sides.

### 3.3. Lifetime Measurement and Optical Characterization

The effective carrier lifetime was measured based on quasi-steady-state photoconductance decay (QSSPC) using a WCT-120 from Sinton Instruments (software version v5.80.3.3), which was calibrated shortly beforehand with a set of calibration wafers. All lifetime measurements were evaluated at a minority carrier density of 1 × 10^15^ cm^−3^.

The thickness of the deposited TiO_2_ layers was measured using ellipsometry (Woollam M-2000, Lincoln, NE, USA) at multiple points across the samples. X-ray photoelectron spectroscopy (XPS) measurements were performed using an ESCALAB 250Xi from Thermo Scientific (Waltham, MA, USA) on 0.5 cm by 0.5 cm samples. The analysis pressure was between 10^−8^ mbar and 10^−9^ mbar with the spot size of the Al Kα (energy hν = 1486.68 eV) source being 500 µm. Grazing Incident X-Ray Diffraction (GIXRD) was performed using a D8 Advance (Bruker Optik GmbH, Billerica, MA, USA) with 2θ between 10° and 100°, a step size of 0.04, and a measurement time of 1 s per step.

## 4. Results

The Bayesian optimization attempt aimed to enhance the surface passivation quality of TiO_2_, deposited using TTIP and water, by identifying sets of processing parameters that yield highly effective carrier lifetimes in symmetrically passivated samples. The search space for the initial optimization attempt was constrained as outlined in Table 1, with the upper and lower bounds primarily determined by the hardware limitations of the ALD system (e.g., valve response times and pressure control capabilities) or reasonable limits based on readily available knowledge of precursor characteristics and chemistry. To ensure that every combination of parameters from within the multidimensional search space was sampled at least once, fourteen quasi-random sets of processing parameters were chosen using Latin Hypercube Sampling with one additional custom parameterization.

**Figure A1:**
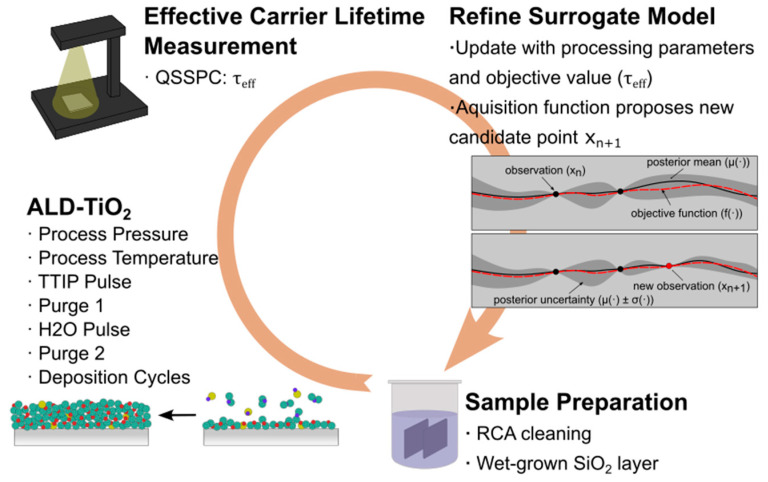
Diagram illustrating a simplified experimental routine during the iterative Bayesian optimization process. After preparing the samples, TiO_2_ is deposited via ALD, with varying key parameters. The effective carrier lifetime is measured and utilized to update the surrogate model. New candidate points are then proposed by the acquisition function, driving subsequent deposition experiments to optimize the surface passivation quality of the deposited TiO_2_ layers.

Each set of processing parameters was then used to symmetrically deposit TiO_2_ on a cleaned substrate, and the resulting effective carrier lifetime was measured using QSSPC. The processing parameters and corresponding effective carrier lifetime of each sample were used to train the optimization algorithm and construct the surrogate model. Based on these initial data points, the sequential optimization algorithm was initiated, which recommended a new set of process parameters to investigate next. The newly suggested set of process parameters was then used to symmetrically deposit TiO_2_, followed by carrier lifetime measurement. The processing parameters and resulting effective carrier lifetime were then used to further update the surrogate model (see Figure A1).

**Figure 1:**
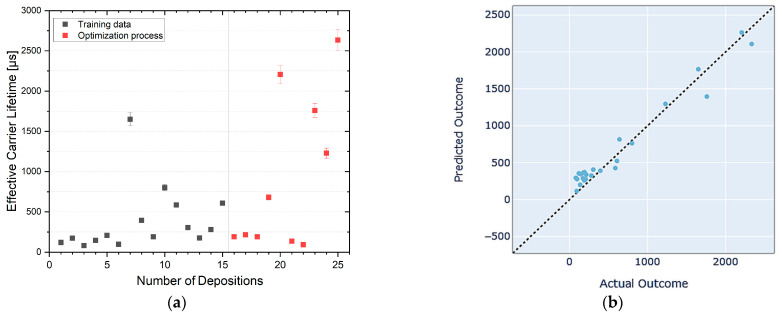
(**a**) Measured effective carrier lifetimes of samples deposited using different sets of processing parameters. Error bars indicate an assumed measurement error of 5% [24]. (**b**) K-fold cross-validation (k = 5) plot of the surrogate model provided by the Ax framework. The dashed line represents perfect predictions. Points clustered closely around the dashed line suggest good model performance, while widespread points suggest areas where the model may need improvement.

For the optimization attempt, we deliberately limited the number of optimization iterations to only ten (see Figure 1a) before interrupting the optimization process and analyzing the surrogate model and the current best set of processing parameters. The sequential nature of the BO process allows for an arbitrary number of optimization iterations, which may be based on the requirements of the optimization goal, resource or time constraints, or diminishing returns in the objective parameter. It is therefore important to note that this specific set of parameters is highly unlikely to already represent the optimized combination of processing parameters that maximizes the surface passivation quality of the TiO_2_ deposition process. Instead, it represents the set of parameters that has yielded the highest surface passivation quality thus far.

Surrogate models can be evaluated through various methods to ensure their accuracy and reliability in approximating the objective function. Among key evaluation techniques are k-fold cross-validation plots, which provide quantitative assessments of the model’s predictive performance [25]. Each point on a cross-validation graph represents a pair of observed and predicted values. An ideal surrogate model would have all points lying on a diagonal line where the predicted values exactly match the observed values. Deviations from this line indicate prediction errors, with the distance from the line reflecting the magnitude of the error.

The cross-validation plot (k = 5) of the current surrogate model (Figure 1b) reveals tight clustering of the data points around the diagonal line denoting ideal model accuracy. No significant deviations were observed, with good agreement between the model’s predictions and the measured effective carrier lifetime, suggesting that the model is generally reliable. However, a closer examination of samples in regions with low effective carrier lifetimes indicates systematic deviations between the predicted and actual outcome. The model tends to overestimate surface passivation quality in low-lifetime regions, resulting in more pronounced discrepancies. Further exploration of this region may be necessary to improve the model’s prediction accuracy in areas where low effective carrier lifetimes are expected. However, given that the primary optimization goal is to identify sets of processing parameters that result in good surface passivation (i.e., highly effective carrier lifetimes), further exploration of the low-lifetime region may not be justified. As the effective carrier lifetimes increase, the model’s predictions align more closely with the actual outcomes, though some deviations towards higher actual surface passivation quality remain, indicating room for improvement in the model’s prediction accuracy. The comparatively low number of samples with high effective carrier lifetimes may not be sufficient to fully capture the behavior of the objective function, emphasizing the need for further optimization iterations.

**Figure 2:**
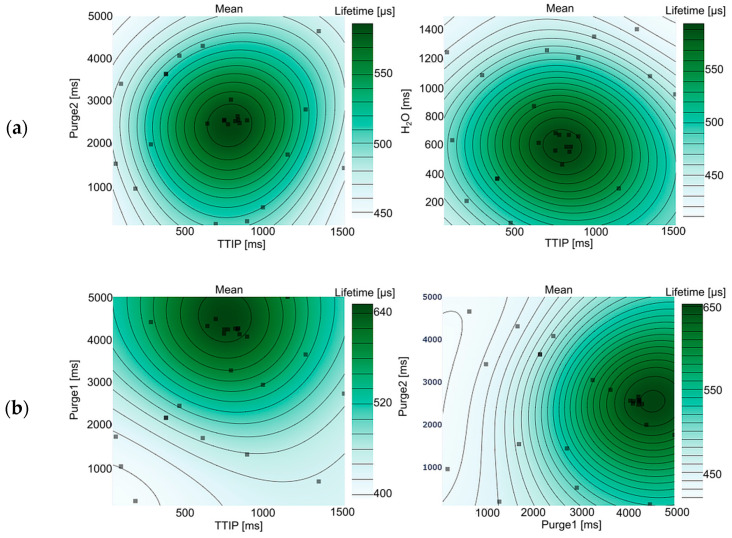

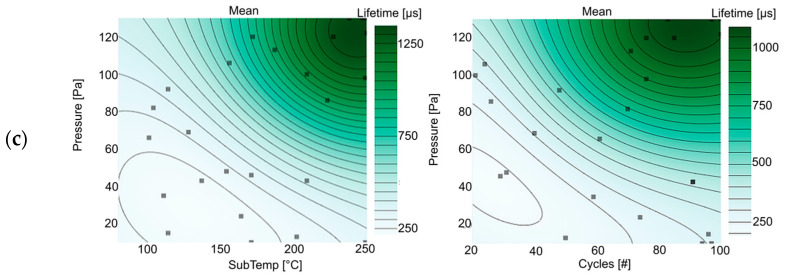
Examples of two-dimensional response surface plots for the mean of the lifetime metric. Showing (**a**) similar influence on the objective parameter, (**b**) a positive impact at high parameter values, (**c**) clustering towards the current parameter constraints. Remaining parameters are fixed to the middle of their respective ranges (see Table 1). The gradient reveals the rate of change in the objective value relative to changes in the input parameters, while the color of the surface shows the magnitude of the objective value.

Other key evaluation methods are response surface plots, which can offer qualitative insights into the surrogate model’s behavior by visualizing predicted values of the objective function across the input space. By varying two input parameters across their ranges (and fixing the remaining input parameters at specific values), the surrogate model can predict the objective function values, which are then plotted to visualize the response surface. Using the Ax framework, the response surface plots of the mean objective value (see Figure 2) and standard error can be visualized.

Different combinations of response surface graphs for the second purge pulse (Purge2) and the dosing times of TTIP and water (H_2_O) show centered symmetrical peaks of high lifetime values (Figure 2a), suggesting that each parameter exerts a similar influence. Notably, response surfaces of the TTIP purge time (Purge1) show higher objective values towards high parameter values (Figure 2b), indicating a positive influence of long purge times after the TTIP precursor step. Response surface graphs of processing pressure, processing temperature, and cycle count exhibit a tendency for high objective values to concentrate near or at the upper limits of these parameters. This is clearly illustrated by plotting the response surface graph of processing pressure versus processing temperature or processing pressure versus cycle count (see Figure 2c). Clustering of high objective values towards the upper (or lower) bound indicates that the respective parameter may be overly constrained, potentially limiting the exploration of regions where more favorable objective values could lie beyond the current boundaries. This assumption is confirmed by comparing the values of the current best set of processing parameters against the upper bounds of the search space set during the design of the experiment (see Table 2).

Processing pressure, processing temperature, and the number of deposited cycles match their respective upper bounds. We can therefore reasonably conclude that the initial search space of the optimization attempt was overly constrained and that there may be combinations of processing parameters beyond the current constraints of the search space that could lead to even better surface passivation quality of the deposited layers. However, not all processing parameters were intentionally limited. The range of processing pressures was solely restricted by hardware limitations, indicating that more optimal solutions may exist beyond the capabilities of the ALD system.

**Figure A2:**
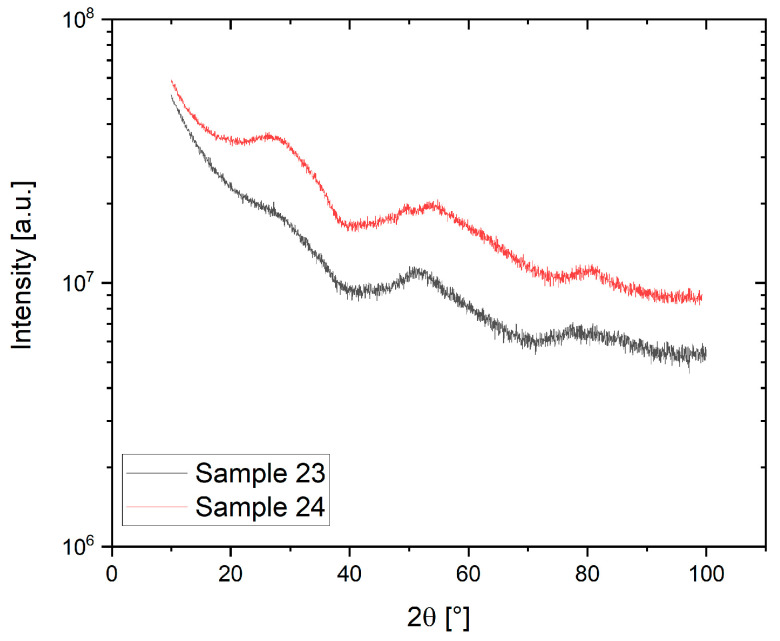
Grazing Incident X-Ray Diffraction (GIXRD) measurements of selected samples deposited using TTIP and water. The lack of narrow and distinct peaks strongly suggests amorphous TiO_2_ layers.

To assess the surface passivation quality of TiO_2_ layers deposited with various processing parameters, five of the ten samples from the iterative optimization process were selected to represent a comprehensive overview. These samples were further characterized using Ellipsometry and XPS measurements. Additionally, Grazing Incident X-Ray Diffraction was performed on selected samples, confirming the amorphous nature of the deposited TiO_2_ layers (see Figure A2). The calculated GPC values (see Table 3) of the samples tend towards the upper range of values usually stated for the deposition of thermal TiO_2_ using TTIP and water (0.15–0.6 Å/cycle) [26], with Sample 23 showing a significantly higher GPC of 0.82 Å/cycle, possibly indicating parasitic CVD. The observed tendency of the optimization algorithm to favor high numbers of deposition cycles suggests a strong influence of the thickness of the deposited layer on the surface passivation quality. However, a comparison of layer thickness and corresponding effective carrier lifetime shows that thicker layers do not generally result in higher effective carrier lifetimes (Table 3).

**Figure A3:**
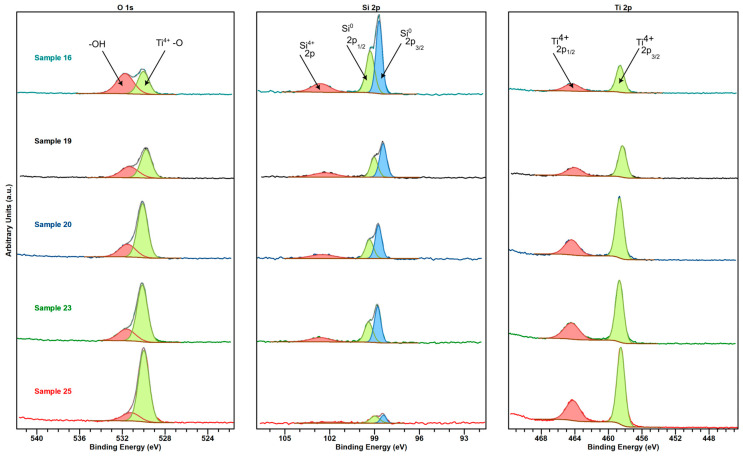
XPS measurements of selected samples deposited during the iterative optimization process. Significant differences in Ti 2p, O 1s, and Si 2p regions between different samples deposited with different processing parameters can be seen.

XPS measurements were performed to determine the relative atomic percentages of constituents within the deposited layers. The relative atom% of Si varies significantly between samples (see Table 4) with no clear relation to the thickness of the overlaying TiO_2_ layer. All samples exhibit two symmetric peaks in the Ti 2p emission spectrum, with the larger peak at around 458.7 eV corresponding to Ti 2p_3/2_, and the smaller peak at around 464.3 eV corresponding to Ti 2p_1/2_, most likely in the 4+ state, according to [27], confirming the deposition of TiO_2_. Closer examination of the Si 2p signals shows asymmetric peaks in all samples with contributions at around 98.6 eV and 99.2 eV, indicating overlapping Si 2p_3/2_ and Si 2p_1/2_ orbitals due to closely spaced spin-orbit components (Δ = 0.63 eV). The binding energy corresponds to Si 2p core levels of elemental Si (Si^0^), a result of the crystalline silicon substrate (Figure A3). A second weaker peak at higher binding energies of around 102.5 eV is present in all samples, indicating the presence of silicon in a fully oxidized state (Si^4+^) with the signal originating from the SiO_2_ layer wet-grown during sample preparation. The O 1s fine spectra show asymmetric peaks with contributions at around 529.8 eV and 531.3 eV. The peaks at lower binding energies are assigned to oxygen atoms bound in the TiO_2_ lattice (Ti-O) and are characteristic of the oxygen in the crystalline or amorphous TiO_2_ phase, with binding energies typically stated as ranging from around 529.6 to 530 eV for TiO_2_ [28]. The observed peaks at around 531.3 eV cannot be assigned to O in SiO_2_, as differences in binding energies of more than 2 eV are expected between O in SiO_2_ and O in TiO_2_ [29]. We assigned these peaks at higher binding energies to oxygen in hydroxyl groups (–OH) and oxygen bound to carbonates [30], with contributions stemming from ambient air contamination and incomplete ligand decomposition of the TTIP precursor during deposition. Carbon contamination is a known issue when depositing TiO_2_ from TTIP and water, and, depending on processing parameters, can significantly affect the purity and properties of the deposited films [31].

Evaluation of the surrogate model and current best set of processing parameters revealed that hardware limitations in processing pressure, as well as low upper bounds in processing temperature and cycle count, have hindered the search for sets of processing parameters capable of achieving even better surface passivation quality. XPS and ellipsometry measurements show that small variations in the number of deposition cycles and the layer thickness appear to be less critical to the resulting surface passivation quality when compared to the composition and the stoichiometry of the deposited layers, which are primarily determined by the other remaining optimization parameters.

## 5. Conclusions

This work demonstrates that freely accessible and easy-to-implement Bayesian optimization software can be effectively leveraged for ALD process optimization. We applied BO to the deposition of thermal ALD thin films, aiming to improve the surface passivation quality of TiO_2_ layers by optimizing seven critical processing parameters: processing pressure, temperature, cycle count, the duration of the first and second purge pulses, and the dosing times of the first and second precursors. A detailed examination of the surrogate model showed good overall prediction accuracy, confirming that BO can effectively guide ALD process optimization. This was further supported by a significant improvement in the surface passivation quality of the deposited TiO_2_ layers after just ten optimization iterations.

Defining an appropriate search space for the algorithm to explore proved challenging, as the initial search space was overly constrained. Hardware limitations or narrow upper bounds on the parameters restricted the search for more optimal processing conditions. Future work could address this by implementing a broader search space and using a more versatile ALD system, allowing for exploration beyond current limitations. Our results also show that the surface passivation quality of ALD-deposited TiO_2_ is not solely dependent on layer thickness but is strongly influenced by layer composition, as evidenced by XPS and ellipsometry measurements. This suggests that ALD process optimization could benefit from exploring parameter spaces beyond the conventional temperature ranges or precursor chemistries commonly found in the literature, potentially uncovering novel deposition regimes with enhanced layer properties.

While this study focuses on improving the surface passivation quality of ALD-TiO_2_, BO can be adapted to guide ALD process optimization for different objectives. Furthermore, our findings indicate that using Bayesian optimization for ALD processes can be particularly advantageous for small-scale experiments and laboratories, where extensive or complex optimizations may be impractical, even with minimal prior knowledge of the deposition process and precursor chemistry.

## Figures and Tables

**Table 1:** Upper and lower bounds of the search space during the optimization attempt.

Search Space	Lower Bound	Upper Bound
Process Pressure [Pa]	10	130
Process Temperature [°C]	90	250
TTIP Pulse [ms]	50	1500
Purge1 Pulse [ms]	50	5000
H_2_O Pulse [ms]	50	1500
Purge2 Pulse [ms]	50	5000
Cycle Count [#]	5	100

**Table 2:** Comparison of the upper bounds of the search space set during the design of the experiment and the set of processing parameters yielding the highest surface passivation quality this far.

Processing Parameter	Upper Bound	Best Parameterization
Process Pressure [Pa]	130	130
Process Temperature [°C]	250	250
TTIP Pulse [ms]	1500	810
Purge1 Pulse [ms]	5000	4250
H_2_O Pulse [ms]	1500	490
Purge2 Pulse [ms]	5000	2540
Cycle Count [#]	100	100

**Table 3:** Number of deposition cycles, layer thickness, calculated GPC, and measured effective carrier lifetimes of selected TiO_2_ layers deposited during the iterative optimization process. The asterisk denotes the current best set of processing parameters.

Sample	Deposited Cycles [#]	Layer Thickness [nm]	Calculated GPC [Å/cycle]	Effective Carrier Lifetime [µs]
25 *	100	6.51 ± 0.037	0.65	2634 ± 132
23	76	6.26 ± 2.797	0.82	1759 ± 88
20	76	4.13 ± 0.01	0.54	2206 ± 110
19	94	5.56 ± 0.505	0.59	640 ± 32
16	96	4.82 ± 0.332	0.50	190 ± 10

**Table 4:** Relative atom % for selected samples deposited during the iterative optimization process. The asterisk denotes the set of processing parameters that yielded the highest effective carrier lifetime so far.

Relative Atom %					
Sample	O 1s	Si 2p	C 1s	N 1s	Ti 2p
25 *	56.96	2.95	17.16	0.55	22.38
23	50.25	12.84	17.75	0.89	18.27
20	47.75	10.98	21.55	2.52	17.20
19	46.48	18.25	19.87	0.60	14.80
16	36.41	25.99	27.74	1.85	8.01

## Data Availability

The datasets presented in this article are not readily available because the data are part of an ongoing study. Requests to access the datasets should be directed to philipp.haeussermann@physik.tu-freiberg.de.
